# The effects of free fatty acid-free bovine serum albumin and palmitate on pancreatic β-cell function

**DOI:** 10.1080/19382014.2025.2479911

**Published:** 2025-03-16

**Authors:** Katherine Sentjens, Renjitha Pillai, Jamie W. Joseph

**Affiliations:** School of Pharmacy, University of Waterloo, Kitchener, ON, Canada

**Keywords:** Islets, palmitate, insulin secretion, albumin, oxygen consumption

## Abstract

Pancreatic β-cells release insulin in response to fluctuations in plasma glucose, amino acids, and free fatty acids (FFA). Clonal cell lines and isolated islets serve as essential early models for studying the impact of nutrients and evaluating potential therapies to address β-cell dysfunction. Acute and chronic changes in FFA levels have been shown to have positive and negative effects on β-cell function both *in vivo* and *in vitro*. A key problem in comparing islet lipid studies from different laboratories is that a wide variety of methods are used to isolate, culture, and assess islet function. The current study compares bovine serum albumin (BSA) types and lipid preparation methods in clonal 832/13 cells and human islets. Changing the percentage and culture conditions when using FFA-free BSA can negatively affect β-cell function compared to regular BSA. Preparing palmitate with FFA-free BSA can rescue insulin secretion compared to treating cells alone with FFA-free BSA. Different methods of preparing palmitate can have unique effects on insulin secretion. Overall, interpreting the effects of lipids on β-cell function is complicated by a number of variables that need to be controlled for in islet experiments.

## Introduction

Type 2 diabetes results from failing β-cell function, and the body’s cells do not effectively respond to insulin. Defects in nutrient-regulated insulin secretion are a key problem in type 2 diabetics; however, how nutrients regulate insulin secretion is incompletely understood. Glucose can stimulate β-cells to secretion insulin by entering the β-cell through glucose transporter 2 and is metabolized by glycolysis and the tricarboxylic acid cycle. The metabolism of glucose increases the production of adenosine triphosphate (ATP) via oxidative phosphorylation.^1–4^ The elevated ATP/ADP ratio leads to ATP-sensitive K^+^-channels closure, plasma membrane depolarization, and activation of voltage-dependent Ca^2+^ channels, resulting in an influx of calcium and insulin secretion.^1–6^

Free fatty acids (FFA) and amino acids are also important regulators of insulin release from β-cells. FFA can enhance glucose’s ability to stimulate insulin secretion.^7,8^ Palmitate has been shown to be the most effective at stimulating acute insulin release.^9^ When cells are exposed to high FFAs chronically, it leads to β-cell dysfunction and lower insulin release.^10,11^ A key first step in regulating FFA effects in β-cells involves the esterification of FFAs, which can directly and indirectly affect insulin secretion.^12^ Understanding the role of FFAs in regulating β-cell function is complicated because there are different FFAs and methods of conjugating FFAs to albumin, and controlling bound and unbound FFAs is not well described.

Several challenges arise when comparing *in vitro* islet lipid studies, particularly regarding the actual concentrations of bound and unbound FFA levels. Bound and unbound FFA levels in the blood are controlled by albumin. Bovine serum albumin (BSA) is commonly used in FFA islet studies. The source of BSA and concentration are variable in islet FFA studies, typically between 0.1–1% (15–151 µM), which is lower than what is seen in the blood (530–758 µM).^13^ Why these BSA concentrations are chosen is not described, but it may be related to the concentration of albumin around islet cells in the interstitial space; however, this has not been determined experimentally. It is also not known whether interstitial space albumin plays a role in controlling the exposure of islet cells to FFAs. It has been estimated that the interstitial space albumin is 111 µM in adipose and 197 µM in skeletal muscle.^14^ These studies suggest that a reasonable estimate for the interstitial albumin concentration around islets is about 151 µM (or 1%), which is a good starting point for planning islet experiments. However, the role of plasma and interstitial space albumin in controlling islet lipid exposure warrants further investigation.

Different types of FFA may exert distinct effects on islet function; however, comparing studies is challenging because non-physiological FFA/BSA molar ratios are sometimes used, and the FFA/albumin molar ratio is not consistently reported in the literature.^15–29^ For instance, details such as the method of conjugating lipids to BSA, the use of stock solutions, and storage duration before use are often not reported. Additionally, high non-physiological FFA/BSA molar ratios are often used in islet studies.^16^ For example, treating INS-1E clonal cells with 0.5 mM palmitate at an FFA/albumin molar ratio of 4.4 or 3.3 resulted in a similar level of apoptosis.^16^ A problem with interpreting these studies is that different percentages and forms of BSA were used. Freshly prepared lipid solutions can lead to more apoptosis than preparing lipids from stock solutions, possibly because of lipid loss during storage.^16^ Complicating matters further is the conflicting data on the effects of FFAs. For example, oleate can lead to similar apoptosis or less apoptosis as compared to palmitate in some studies, and the only difference between these studies is how the lipids are prepared.^16^ These studies suggest that the form of BSA and the method of lipid preparation can impact the results.

Another potential factor contributing to the effects of FFA in isolated islets is the use of fetal bovine serum (FBS) or another serum in the culture media. The use of FBS in the culture media while islets are being treated with chronic high fat will add more albumin and other factors. For example, FBS has around 2–4% albumin and has FFA, cholesterol, chylomicrons, very low-density lipoproteins (VLDL), low-density lipoproteins (LDL), high-density lipoproteins (HDL), and other factors.^10,30^ At a typical value of 10% FBS in the islet culture media will increase the albumin concentration, and FFAs in the experimental media.^30^ Also, lipoprotein lipase expression in islet cells will release the FBS FFAs from triglycerides (TG) stored in chylomicrons and VLDL.^31–33^ The amount of FFA released from these sources is unknown and may be variable depending on the batch of FBS used and the level of lipoprotein lipase expression.

Not all studies provide enough information on how they prepared their FFAs.^18–27,34–37^ This study compares different BSA types and lipid preparation methods using clonal 832/13 cells and human islets. Our findings demonstrate that the percentage and culture conditions of FFA-free BSA can independently influence outcomes compared to regular BSA. We also show that lipid preparation methods can impact lipid solubility and glucose-stimulated insulin secretion.

## Methods

### Reagents

Unless otherwise specified, all reagents and kits were obtained from SIGMA (St Louis, MO).

### Preparation of FFAs

Method 1: Involves dissolving 63 mg of sodium palmitate (SIGMA P9767) in 1 mL of H_2_O at 70°C for 1 hour (sodium palmitate concentration of 226 mM). 100 µL was transferred to 0.9 mL of a pre-warmed KRB with 5% FFA-free BSA (SIGMA A8806) (sodium palmitate concentration of 22.6 mM). This solution was then probe-sonicated and incubated for 30–60 minutes at 50°C. Finally, 1 mL of 5% FFA-free BSA in KRB with palmitate was transferred to 15 mL of pre-warmed KRB without BSA and incubated for another 30–60 minutes at 50°C. The calculated final concentration of sodium palmitate was 1.4 mM. This solution was then filtered to remove any large particles of palmitate that did not dissolve, and the pH was corrected to 7.4. The FFA level of the resulting buffer was measured using a WAKO-NEFA kit, which gave an average measured concentration of 0.201 ± 0.005 mM sodium palmitate and a final concentration of FFA-free BSA of 0.31%. Controls were prepared using the same method without palmitate. This palmitate solution was used for some experiments. This preparation method was tested with different solvents and forms of palmitate to try to increase the measured FFA concentration of the solution (Table 1).

**Table 1.** Average measured palmitate concentrations (using a Wako FFA kit) obtained using method 1 and various forms of palmitate and initial solvents (water, ethanol, sodium hydroxide, or 5% FFA free BSA in KRB). All palmitate solutions were prepared at a final calculated concentration of 1.4 mM and an FFA-free BSA concentration of 0.31%. Palmitic acid SIGMA P5585. The combination of sodium palmitate and water was the most effective (*n* = 5).

Form of Palmitate	Initial Solvent	Measured Palmitate concentration
Palmitic acid	Water	Did not dissolve
Palmitic acid	Ethanol	156 ± 6 µM
Palmitic acid	Sodium hydroxide	169 ± 11 µM
Palmitic acid	5% FFA free BSA in KRB	Did not dissolve
Sodium palmitate	Water	201 ± 5 µM
Sodium palmitate	Ethanol	Did not dissolve
Sodium palmitate	Sodium hydroxide	165 ± 12 µM
Sodium palmitate	5% FFA free BSA in KRB	128 ± 8 µM

Sodium Palmitate preparation method 2: The second preparation method involved dissolving 30.6 mg of sodium palmitate in 44 mL of 150 mM sodium chloride at 70°C while stirring in a glass beaker for 1 hour (palmitate concentration 2.5 mM). 40 mL of this solution was transferred to 50 mL of a pre-warmed 0.62% FFA-free BSA KRB solution while stirring (palmitate concentration 1.0 mM). The palmitate solution was then adjusted to 100 ml to match the concentrations of the KRB buffer and then incubated for 1 hour at 40°C. The solution was filtered and corrected to a pH of 7.4 (the final calculated palmitate concentration was 0.91 mM). When the FFA level was measured using a WAKO-NEFA kit, the average sodium palmitate concentration was determined to be 0.431 ± 0.007 mM with a final FFA-free BSA concentration of 0.31%. Controls were prepared using the same method without palmitate.

### Cell lines and insulin secretion assay

The 832/13 cell line,^38^ derived from INS-1 rat insulinoma cells, was a kind gift from C.B. Newgard.^39^ Culture and insulin secretion assay was performed as previously described.^40–44^ Krebs Ringer Bicarbonate (KRB): NaCl 129 mM, KCl 4.38 mM, MgSO_4_ *7 h_2_O 1.2 mM, KH_2_PO_4_ 1.5 mM, HEPES 10 mM, HCO_3_ 5 mM, CaCl_2_ 3.11 mM, pH 7.4.

### Oxygen consumption

Oxygen consumption was measured using an XF24 extracellular flux analyzer in 832/13 cells (Seahorse Bioscience, Billerica, MA).^45^ For Seahorse experiments, the KRB was modified by adding 1 mM of Na_2_HPO_4_, HEPES was reduced to 2 mM, and no HCO_3_ was added. On the day of the assay, cells were pre-treated with KRB containing 2 mM glucose for 2 h with no BSA or CO_2_. Oxygen consumption was then measured in response to varying concentrations of glucose plus or minus palmitate; 5 µM oligomycin; 50 µM 2,4-dinitrophenol (DNP) with 20 mM pyruvate for cells; 5 µM rotenone and 5 µM myxothiazol. Injection of these drugs was used to analyze cellular oxygen consumption. Oxygen consumption associated with ATP turnover was determined as the difference between oxygen consumption in response to oligomycin and glucose. Proton leak across the inner mitochondrial membrane was assessed as the difference between oxygen consumption in response to oligomycin and rotenone + myxothiazol. DNP and pyruvate were used to determine the maximum respiratory capacity of the cells. Rotenone and myxothiazol were also used to assess oxygen consumption, which was not associated with the electron transport chain.

### Human islets

Human islets were provided by the Alberta Diabetes Institute Islet Core at the University of Alberta in Edmonton, with the assistance of the Human Organ Procurement and Exchange Program and the Trillium Gift of Life Network in the procurement of donor pancreata for research (islets from 9 donors with an average age of 58, See supplemental Table S1 for donor information).^46,47^

### Human islet insulin secretion assay

Insulin secretion in response to glucose was measured as described previously.^40–44^ The islet KRB buffer used for insulin secretion assays consisted of 120 mM NaCl, 4.8 mM KCl, 2.5 mM CaCl_2_, 1.2 mM MgCl_2_, 5 mM NaHEPES, and 24 mM NaHCO_3_ at pH 7.4. BSA was added to KRB as described in the results section. Briefly, islets were pretreated for 1 h in KRB with 2 mM glucose, then treated for 1 h in KRB containing glucose plus or minus palmitate. After the assay, the buffer was collected and assayed for insulin by a radioimmunoassay kit (SIGMA).

### Statistical analysis

**Figure 1.**
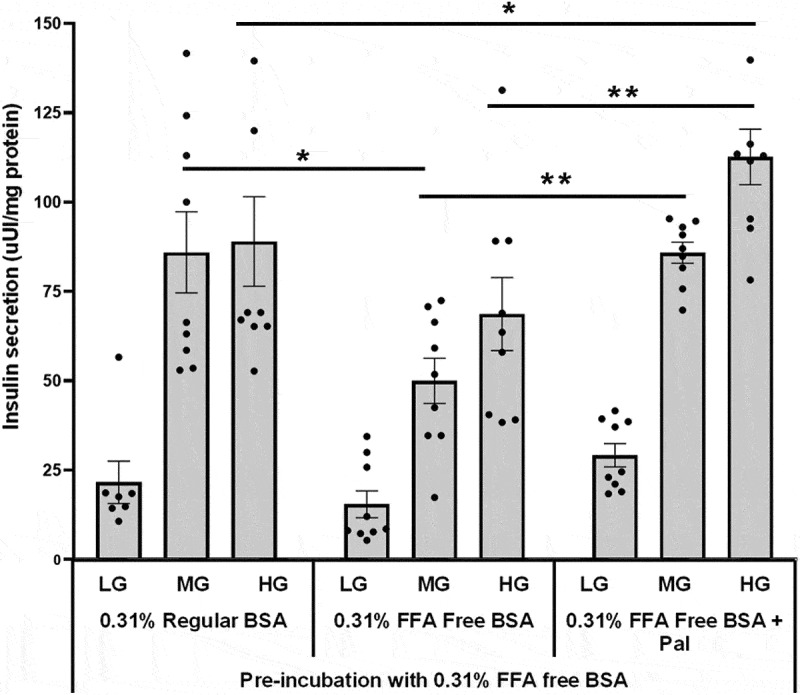
The effects of regular or FFA-free BSA on glucose- and palmitate-stimulated insulin secretion in 832/13 cells. 832/13 cells were pretreated with 0.31% FFA-free BSA for 2 hours at 2 mM glucose. Cells were then treated for 2 hours with 2 mM low glucose (LG), 6 mM medium glucose (MG), or 16.7 mM high glucose (HG) with either regular or FFA-free BSA plus or minus palmitate. BSA concentrations were 0.31% for all treatments. Sodium palmitate was prepared using lipid preparation method 1 at 1.4 mM with a final measured concentration of 200 µM. The palmitate to BSA ratio was 4.3:1 (*n* = 9). *p < 0.05, **p < 0.01, ***p < 0.001.

**Figure 2.**
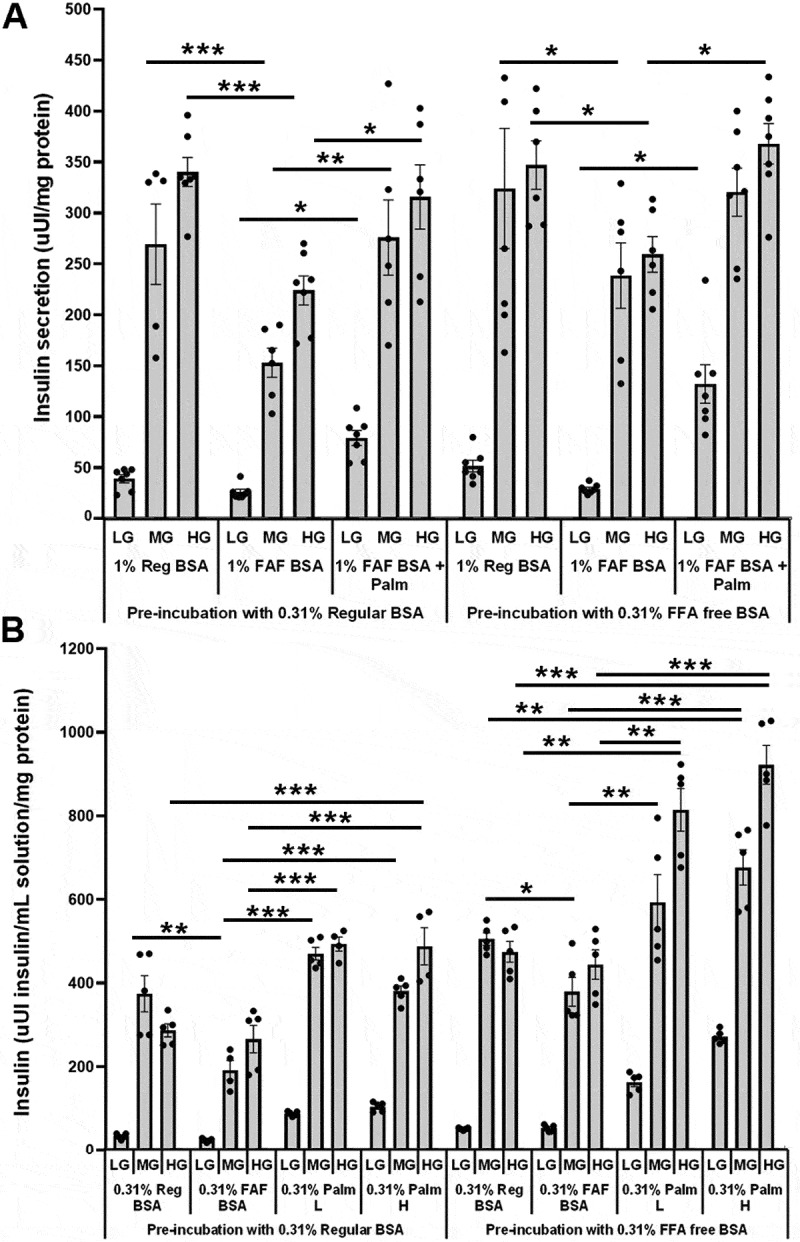
The effects of pretreating 832/13 cells for 2 hours with 2 mM glucose and either 0.31% regular or FFA-free BSA on glucose- and lipid-stimulated insulin secretion. A) During the secretion assay, the percent BSA was increased to 1% for all treatments compared to 0.31% in Figure 1. Cells were treated with LG, MG or HG. Sodium palmitate was prepared using lipid preparation method 1 at 1.4 mM with a final measured concentration of 200 µM. The palmitate to BSA ratio was 1.32:1 (*n* = 5–7). B) the effects of changing the lipid preparation method on glucose- and lipid-stimulated insulin secretion in 832/13 cells. During the pretreatment and secretion assay, the percent BSA was 0.31%. Cells were treated with LG, MG or HG. Sodium palmitate was prepared using lipid preparation method 2 at 1.1 mM with a final measured concentration of 400 µM (Palm H). This lipid preparation was diluted to 200 µM for some experiments (Palm L). The palmitate to BSA ratio was 4.27:1 (200 µM, Palm L) and 8.55:1 (400 µM, Palm H) (*n* = 4–5). *p < 0.05, **p < 0.01, ***p < 0.001.

All results are given as mean ± SEM. Statistical significance was assessed using GraphPad Prism version 10. Figures 1, 2, 7–9 were analyzed using a 2-way ANOVA followed by Holm-Šídák’s multiple comparisons test. There was no significant interaction between BSA treatments and the different glucose concentrations. Spearman’s test was used to assess heteroscedasticity, and D’Agostino-Pearson omnibus (K2) and QQ plots were used to determine the normality of residuals. For Figure 2, statistics were only performed within each preincubation grouping and not between the preincubation treatments.

## Results

### The effects of FFA-free BSA and sodium palmitate on insulin secretion

832/13 clonal β-cells were first pretreated with 0.31% FFA-free BSA (46.8 µM BSA concentration) for 2 hours, followed by assessing glucose-stimulated insulin secretion with either regular BSA, FFA-free BSA or FFA-free BSA plus 200 µM sodium palmitate (palmitate to BSA ratio of 4.3:1). The sodium palmitate KRB was prepared using method 1. No significant differences were seen between treatments when cells were incubated with low glucose (LG, 2 mM) (Figure 1). When the glucose concentration was increased to 6 mM, incubating cells with FFA-free BSA significantly inhibited insulin secretion, which was rescued by incubating cells with FFA-free BSA plus 200 µM sodium palmitate. At 16.7 mM, incubating cells with FFA-free BSA plus 200 µM sodium palmitate significantly enhanced insulin secretion compared to cells treated with regular and FFA-free BSA (Figure 1). These studies show that the 16.7 mM glucose plus palmitate enhanced insulin secretion over 16.7 mM glucose alone, irrespective of the type of BSA. In contrast, palmitate with 6 mM glucose could only enhance insulin secretion compared to cells incubated with FFA-free BSA. These results also suggest that 0.31% FFA-free BSA may deplete cells of a critical factor important for regulating insulin secretion at intermediate glucose concentrations.

### The effects of treating 832/13 cells with 1% BSA

To investigate whether increasing the BSA concentration to 1% affects insulin secretion, cells were first preincubated with either 0.31% regular or FFA-free BSA for 2 hours. Cells were then treated with 1% regular or FFA-free BSA and varying glucose concentrations. No major differences existed between the effects of pretreating cells with either 0.31% regular or FFA-free BSA (Figure 2A). Pretreating cells with 0.31% regular BSA or FFA-free BSA followed by 1% FFA-free BSA significantly inhibited insulin secretion at medium glucose (MG, 6 mM) and high glucose (HG, 16.7 mM) glucose concentrations as compared to 1% regular BSA (Figure 2A). Pretreating cells with 0.31% regular BSA followed by incubating cells with 200 µM sodium palmitate (prepared using method 1) with 1% FFA-free BSA at a palmitate to BSA ratio of 1.3:1 rescued the MG and HG-stimulated insulin secretion as compared to cells treated with MG and HG plus 1% FFA-free BSA (Figure 2A). Similar results were found for cells pretreated with 0.31% FFA-free BSA, except for the ability of MG plus palmitate to rescue insulin secretion compared to MG plus FFA-free BSA, which did not reach significance (Figure 2A). Pretreating cells with either regular or FFA-free BSA followed by palmitate also increased LG-stimulated insulin secretion compared to LG FFA-free BSA-treated cells (Figure 2A). These results suggest that increasing the FFA-free BSA concentration led to an inhibition of insulin secretion, and this was not affected by pretreating with FFA-free BSA or regular BSA. These results also suggest that reducing the unbound palmitate levels by changing the ratio of palmitate to BSA to 1.3:1 did not affect palmitate’s ability to rescue insulin secretion but did affect the ability of palmitate to enhance insulin secretion as compared to regular BSA. These results also suggest that preincubating cells with FFA-free BSA did not significantly affect the ability of 832/13 cells to secrete insulin in response to glucose with regular BSA, which means the ability of FFA-free BSA to affect insulin secretion occurs only during the treatment period.

### The effects of changing the sodium palmitate preparation method on insulin secretion

When the preparation method of sodium palmitate was changed to method 2, it increased the solubility of palmitate and allowed for the preparation of higher concentrations of lipids. All pretreatments and secretion assays were performed with 0.31% regular or FFA-free BSA concentration. Pretreating cells with regular BSA or FFA-free BSA followed by treating them with FFA-free BSA in the secretion assay buffer inhibited glucose-stimulated insulin secretion compared to treating them with regular BSA when treating cells with 6 mM glucose (Figure 2B). Pretreating cells with regular BSA followed by treating them with 200 µM or 400 µM sodium palmitate significantly enhanced insulin secretion compared to treating them with FFA-free BSA (Figure 2B). Interestingly, pretreating cells with FFA-free BSA followed by treating with FFA-free BSA with either 200 µM or 400 µM sodium palmitate enhanced glucose-stimulated insulin secretion compared to regular and FFA-free BSA (Figure 2B). The increased insulin secretion with palmitate vs. regular BSA did not occur using method 1 preparation of sodium palmitate, as seen in Figure 1, suggesting the method of palmitate preparation can affect the ability of cells to respond to palmitate. These results also indicate that preincubating cells with 0.31% FFA-free BSA and increasing the palmitate-to-BSA ratio can significantly impact the ability of palmitate to stimulate insulin secretion. The ability of FFA-free BSA to affect insulin secretion also depends on the concentration of BSA in the secretion assay.

### The metabolic effects of FFA-free BSA on insulin secretion

**Figure 3.**
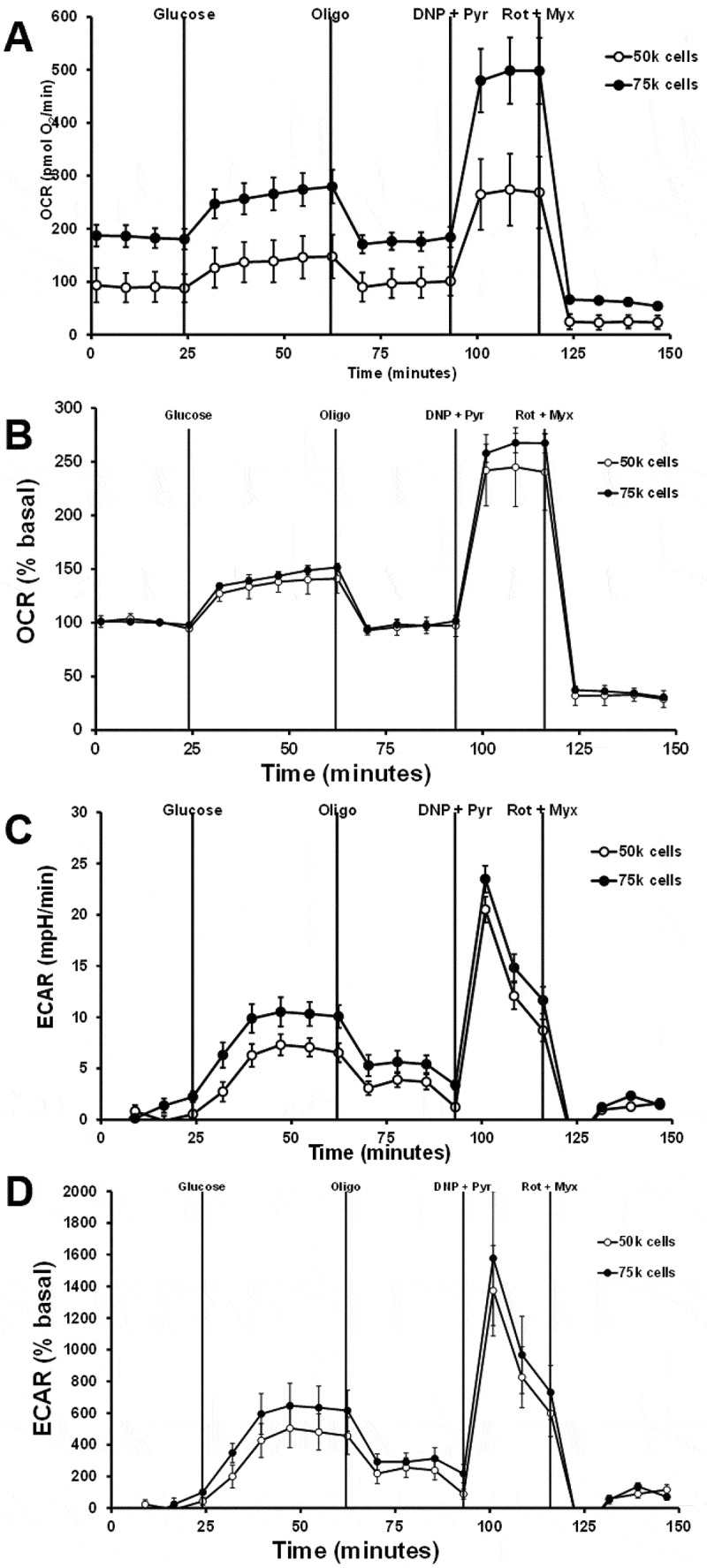
The effects of cell number on the OCR and ECAR responses. 832/13 cells were plated at a density of 50,000 or 75,000 cells per well. Cells were pretreated with LG without BSA for 2 hours. Cells start with LG with 0.1% regular BSA. The sequence of injections is high glucose (Glucose, 10 mM), oligomycin (Oligo, 20 µM), dinitrophenol (DNP, 100 µM) plus pyruvate (Pyr, 20 mM), rotenone (Rot, 10 µM) plus myxothiazol (Myx, 10 µM). A) Raw OCR values. B) Percent basal OCR values. C) Raw ECAR values. D) Percent basal ECAR values (*n* = 8).

Seahorse was used to assess the effects of changing cell density, glucose concentration, BSA type, and palmitate on oxygen consumption rate (OCR, a measure of mitochondrial respiration) and extracellular acidification rate (ECAR, a measure of glycolytic rate). 832/13 cells were plated at a density of 50,000 or 75,000 cells per well, and then five days later, the plates were assessed using Seahorse. Cells were first preincubated with KRB without BSA for 2 hours. BSA was not included in the preincubation assay buffer since Seahorse did not recommend it. Also, BSA can interfere with the effectiveness of some of the hydrophobic drugs used in the assay. OCR was higher for cells plated at the higher density for all measurements, but when the OCR was corrected percent basal, both cell densities showed a similar response (Figure 3A,B). ECAR responses were higher for plates seeded at a higher density for either raw data or data corrected for percent basal (Figure 3C,D).

**Figure 4.**
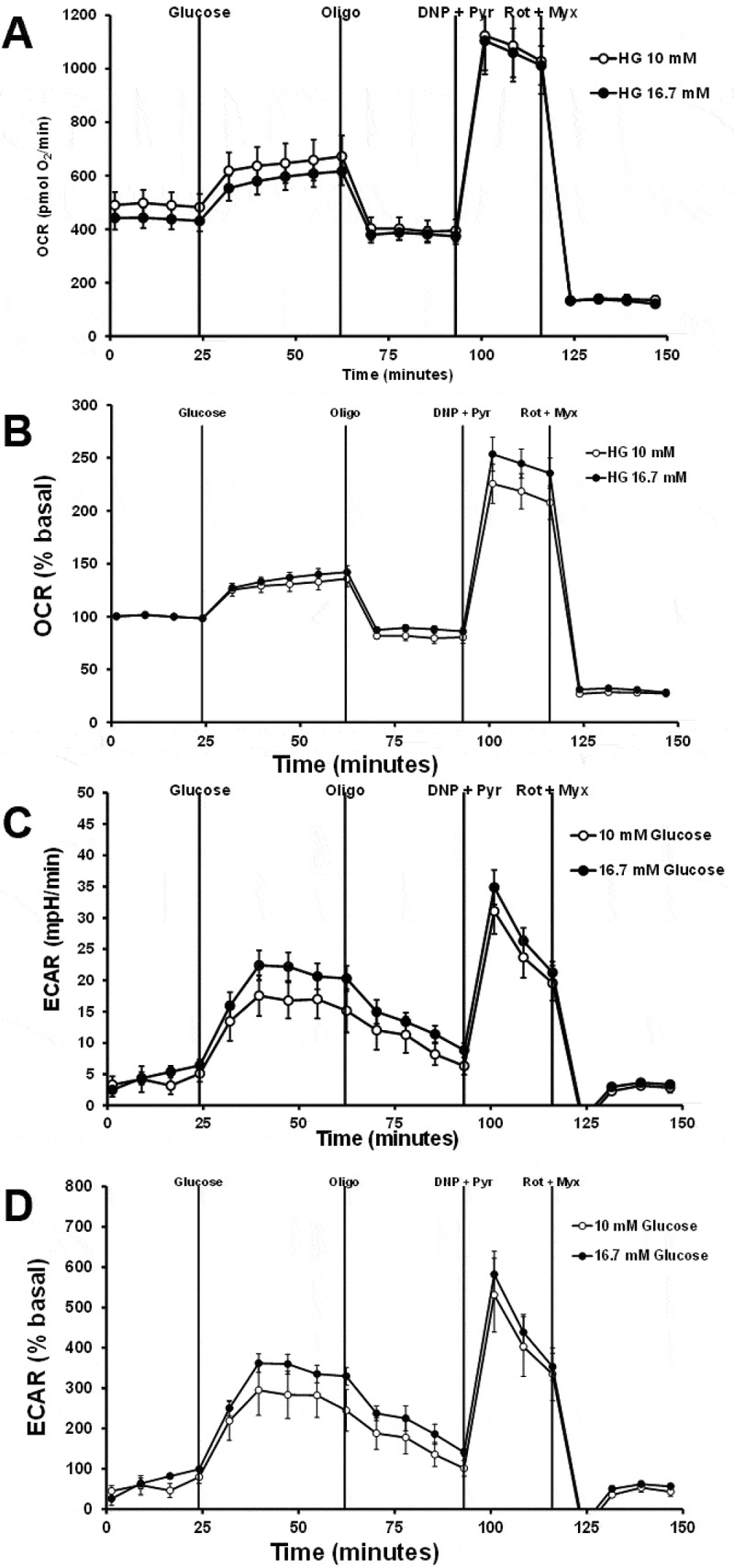
The effects of glucose concentration on the OCR and ECAR responses. 832/13 cells were plated at a density of 50,000 cells per well. Cells were pretreated with LG without BSA for 2 hours. Cells start with LG with 0.1% regular BSA. The sequence of injections is high glucose (glucose, 10 mM or 16.7 mM), oligomycin (Oligo, 20 µM), dinitrophenol (DNP, 100 µM) plus pyruvate (Pyr, 20 mM), rotenone (Rot, 10 µM) plus myxothiazol (myx, 10 µM). A) Raw OCR values. B) Percent basal OCR values. C) Raw ECAR values. D) Percent basal ECAR values (*n* = 8).

**Figure 5.**
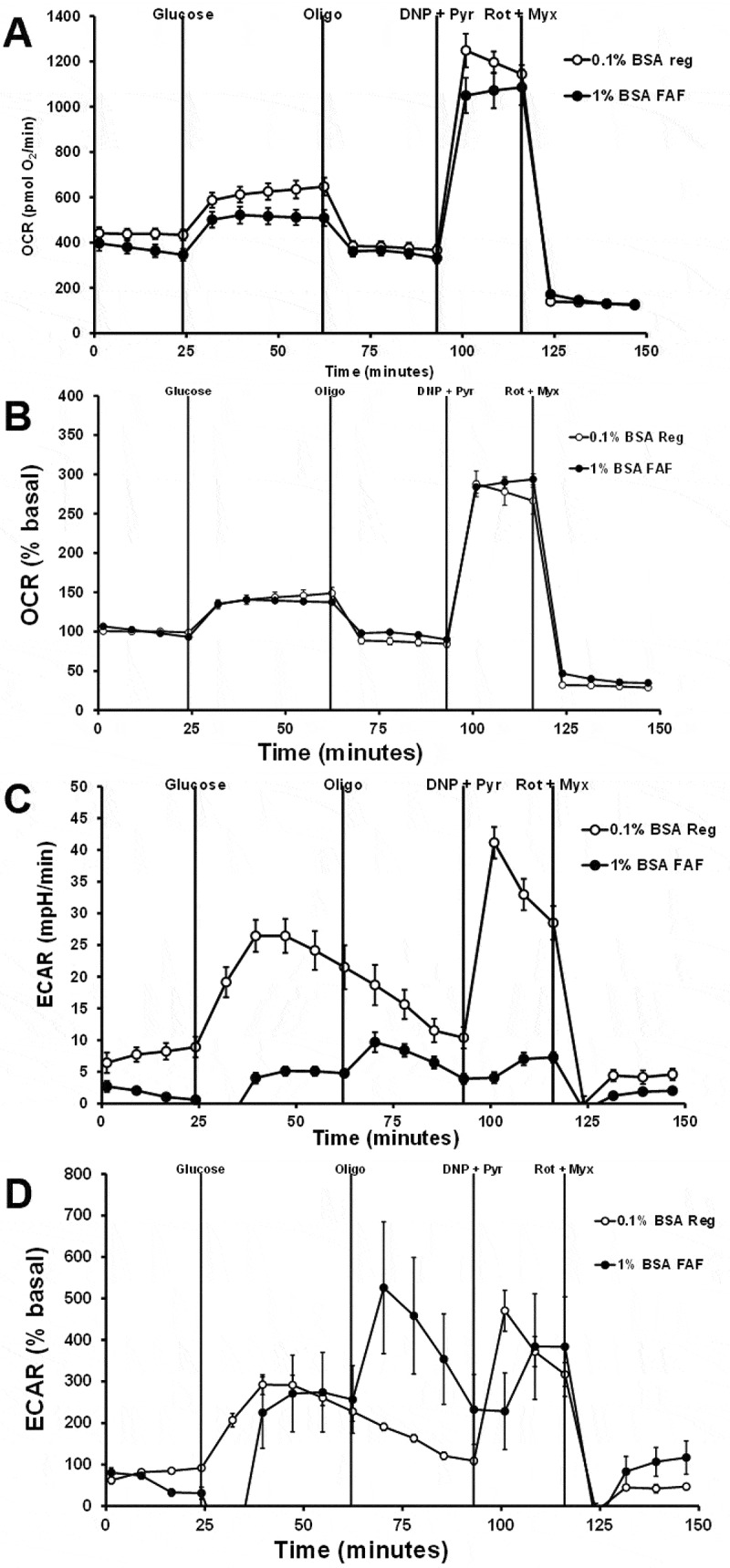
The effects of 0.1% regular BSA vs. 1% FFA-free BSA on OCR and ECAR responses. 832/13 cells were plated at 50,000 cells per well. Cells were pretreated with LG without BSA for 2 hours. Cells start with LG with 0.1% regular BSA or 1% FFA-free BSA. The sequence of injections is high glucose (Glucose, 10 mM), oligomycin (Oligo, 20 µM), dinitrophenol (DNP, 100 µM) plus pyruvate (Pyr, 20 mM), rotenone (Rot, 10 µM) plus myxothiazol (Myx, 10 µM). A) Raw OCR values. B) Percent basal OCR values. C) Raw ECAR values. D) Percent basal ECAR values (*n* = 8).

With a cell density of 50,000 cells, the effects of glucose concentration on the OCR and ECAR responses were assessed. Cells were pretreated with KRB without BSA for 2 hours. Raw and percent basal OCR responses were not different between 10 and 16.7 mM glucose (Figure 4A,B). Raw and percent basal ECAR was higher for cells treated with 16.7 mM glucose than with 10 mM glucose (Figure 4C,D). Changing the BSA type and concentration from 0.1% regular BSA to 1% FFA-free BSA reduced raw OCR values but did not affect percent basal OCR responses (Figure 5A,B). Increasing to 1% FFA-free BSA affected ECAR values, giving variable responses and at times appeared to fall below the assay’s detection limit (Figure 5C,D). These data suggest that 1% FFA-free BSA may affect the probe’s ability to sense pH changes. These data show that changing cell density, glucose concentration, or BSA concentration does not significantly affect OCR responses but does affect ECAR responses. These data also suggest that FFA-free BSA can affect raw OCR data, but this can be corrected for percent basal OCR.

### The effects of palmitate on OCR and ECAR

**Figure 6.**
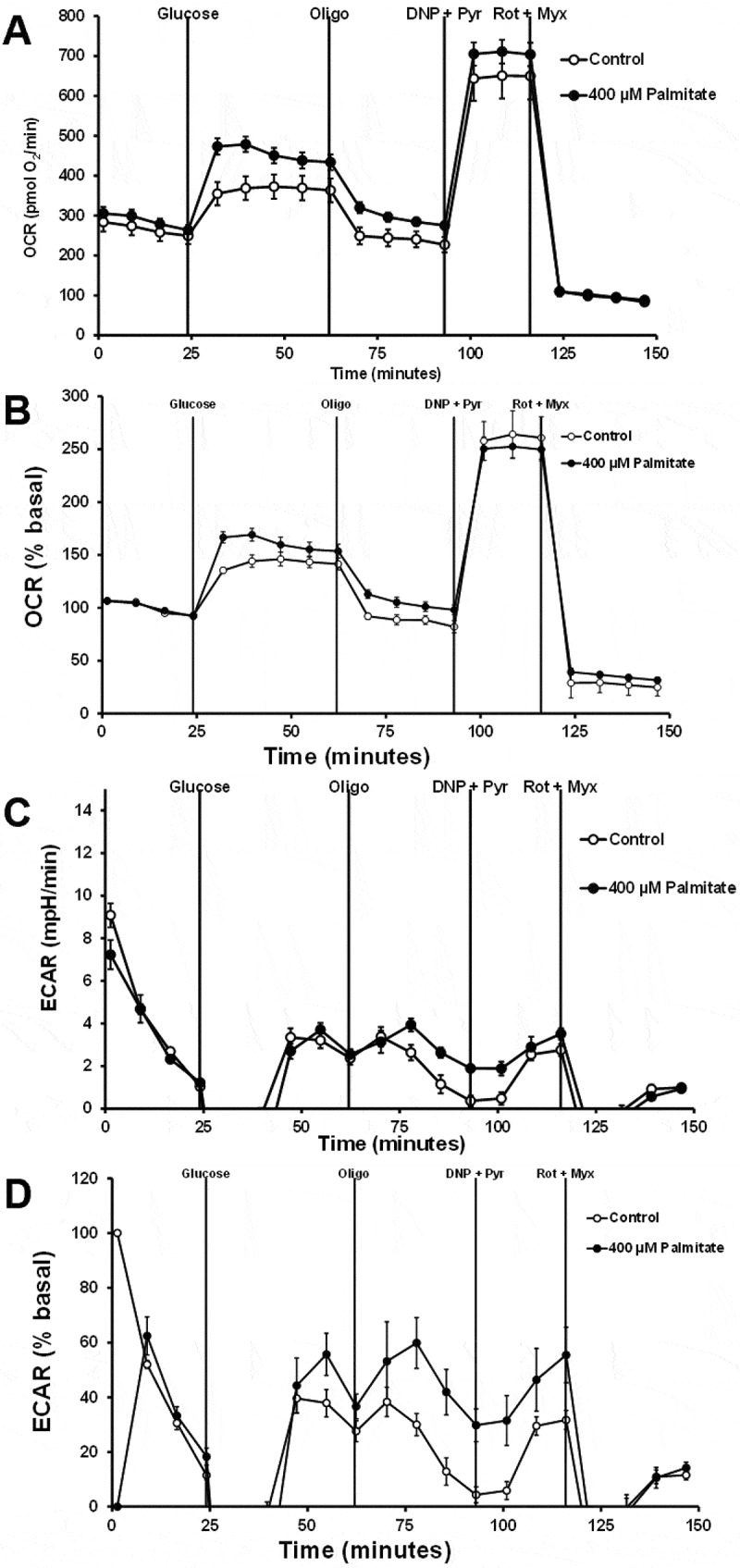
The effects of 1% FFA-free BSA with and without sodium palmitate on the OCR and ECAR responses. 832/13 cells were plated at 50,000 cells per well. Cells were pretreated with LG without BSA for 2 hours. Cells start with LG with 1% FFA-free BSA. The sequence of injections is high glucose (Glucose, 10 mM ± palmitate, 400 µM), oligomycin (Oligo, 20 µM), dinitrophenol (DNP, 100 µM) plus pyruvate (Pyr, 20 mM), rotenone (Rot, 10 µM) plus myxothiazol (Myx, 10 µM). The palmitate to BSA ratio was 2.6:1. A) Raw OCR values. B) Percent basal OCR values. C) Raw ECAR values. D) Percent basal ECAR values (*n* = 15).

Cells were plated at a density of 50,000 cells per well and five days later assessed. Cells were pretreated with KRB without BSA for 2 hours, and the assay buffer contained 1% FFA-free BSA. Since a concentrated stock of sodium palmitate was needed in the injection port of the Seahorse system, only method 2 could be used to prepare sodium palmitate. Sodium palmitate was injected into the well at a final measured concentration of 400 µM with a physiological palmitate to BSA ratio of 2.6:1. When 10 mM glucose plus 400 µM palmitate was added to cells, it increased raw and percent basal OCR levels (Figure 6A,B). Incubating cells with 1% FFA-free BSA with and without palmitate resulted in variable ECAR responses (Figure 6C,D) and were inconsistent with the results seen when cells were treated with 0.1% regular BSA, as seen in Figure 5C,D. These data indicate that 400 µM palmitate enhanced the OCR compared to 1% FFA-free BSA alone. These data also indicate that Seahorse ECAR measurements are not possible when using 1% FFA-free BSA.

**Figure 7.**
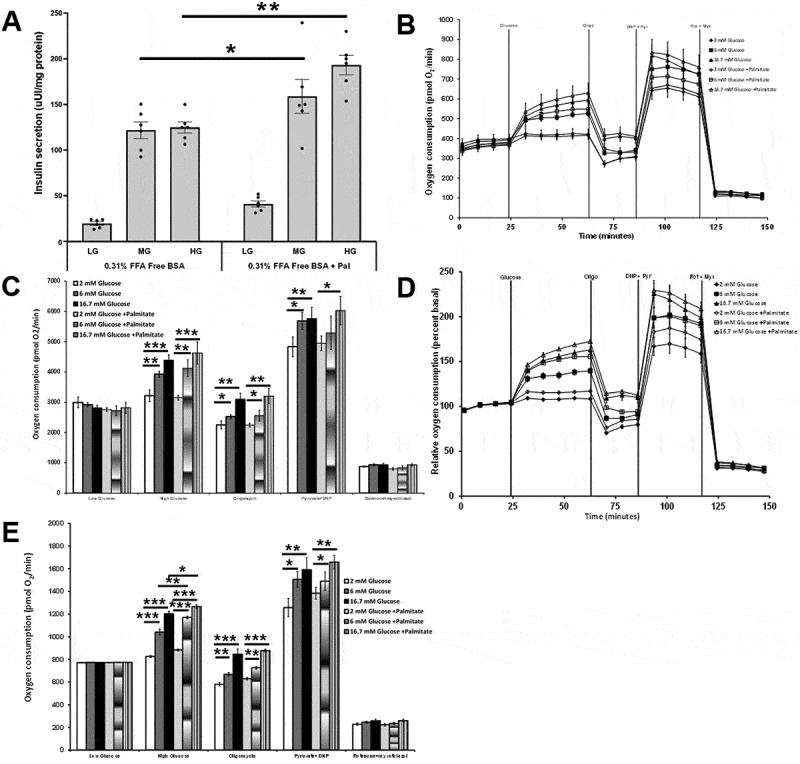
The effects of glucose and palmitate on insulin secretion and OCR responses. 832/13 cells were plated at 50,000 cells per well. Cells were pretreated with LG without BSA for 2 hours. A) Insulin secretion. LG 2 mM, MG 6 mM, or HG 16.7 mM ± palmitate, 50 µM) B) Raw OCR values. Cells start with LG with 0.31% FFA-free BSA (0 minutes). The sequence of injections is glucose (either LG, MG or HG) ± 50 µM palmitate at 25 minutes, oligomycin (Oligo, 20 µM) at 65 minutes, dinitrophenol (DNP, 100 µM) plus pyruvate (Pyr, 20 mM) at 85 minutes, rotenone (Rot, 10 µM) plus myxothiazol (Myx, 10 µM) at 120 minutes. The palmitate to BSA ratio was 1:1. C) area under the curve of B. D) Percent basal OCR values. E) Area under the curve of D (*n* = 18). *p < 0.05, **p < 0.01, ***p < 0.001.

Pretreating 832/13 cells with 0.31% FFA-free BSA for 2 hours, followed by treating with 0.31% FFA-free BSA and varying concentrations of glucose and palmitate, was assessed next. These experiments were performed with a sodium palmitate concentration of 50 µM and a palmitate to BSA ratio of 1:1. Compared to no palmitate, increasing the glucose concentration in the presence of 50 µM palmitate enhanced insulin secretion at both 6 mM and 16.7 mM glucose (Figure 7A). The raw and percent basal OCR responses showed that increasing the glucose concentration from 2 mM to 16.7 mM dose-dependently increased the OCR (Figure 7B–E). Incubating cells with increasing concentrations of glucose with 50 µM palmitate led to a slight increase in raw OCR at 6 and 16.7 mM glucose levels as compared to glucose alone, but it did not reach significance (Figure 7B–C). When the raw OCR responses were corrected to percent basal, increasing concentrations of glucose with 50 µM palmitate led to a significant increase in OCR at 6 and 16.7 mM glucose levels compared to glucose alone, with the response at 6 mM glucose plus palmitate being more significant than the response at 16.7 mM plus palmitate (Figure 7D–E). These results suggest that palmitate’s ability to enhance oxygen consumption is best at an intermediate glucose concentration.

**Figure 8.**
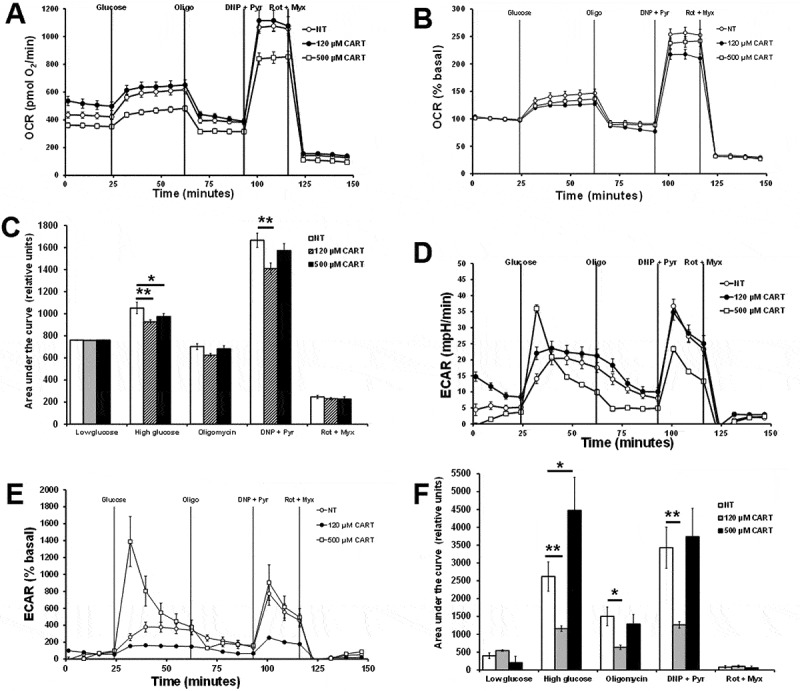
The effects of carboxyatractyloside (CATR) on OCR and ECAR responses. Cells were pretreated with LG without BSA for 2 hours. 832/13 cells were plated at 50,000 cells per well. Cells start with LG with 0.1% regular BSA. The sequence of injections is high glucose (Glucose, 10 mM ± CATR, 120 or 500 µM), oligomycin (Oligo, 20 µM), dinitrophenol (DNP, 100 µM) plus pyruvate (Pyr, 20 mM), rotenone (Rot, 10 µM) plus myxothiazol (Myx, 10 µM). A) Raw OCR values. B) Percent basal OCR values. C) area under the curve of B. C) Raw ECAR values. D) Percent basal ECAR values. F) area under the curve of E (*n* = 18–20). *p < 0.05, **p < 0.01.

The ATP/ADP carrier inhibitor carboxyatractyloside (CATR) was assessed for its effects on OCR and ECAR responses in 832/13 cells. Cells were pretreated with KRB without BSA for 2 hours. After correcting the baseline of the raw OCR (Figure 8A), when cells were treated with 10 mM glucose, the percent basal OCR was significantly inhibited by increasing concentrations of CATR (120 or 500 µM) (Figures 8B,C). After correcting the baseline raw ECAR data (Figure 8D), 120 µM CATR significantly inhibited ECAR, whereas 500 µM CATR led to a significant increase in ECAR (Figures 8E,F). These data suggest that blocking mitochondrial ATP/ADP transport inhibits oxygen consumption at both CATR concentrations and the glycolytic rate was reduced at 120 µM CATR and increased glycolytic rate at 500 µM CATR. The reduced oxygen consumption was expected due to feedback inhibition due to the lack of ATP transport from mitochondria. The lower CATR concentration likely inhibited the glycolytic rate due to the feedback lower mitochondria TCA cycle activity and pyruvate transport. The increased glycolytic rate at the higher CATR was not expected but may be due to increased anaerobic respiration.

### The effects of acute and chronic treatment of human islets with glucose and palmitate

**Figure 9.**
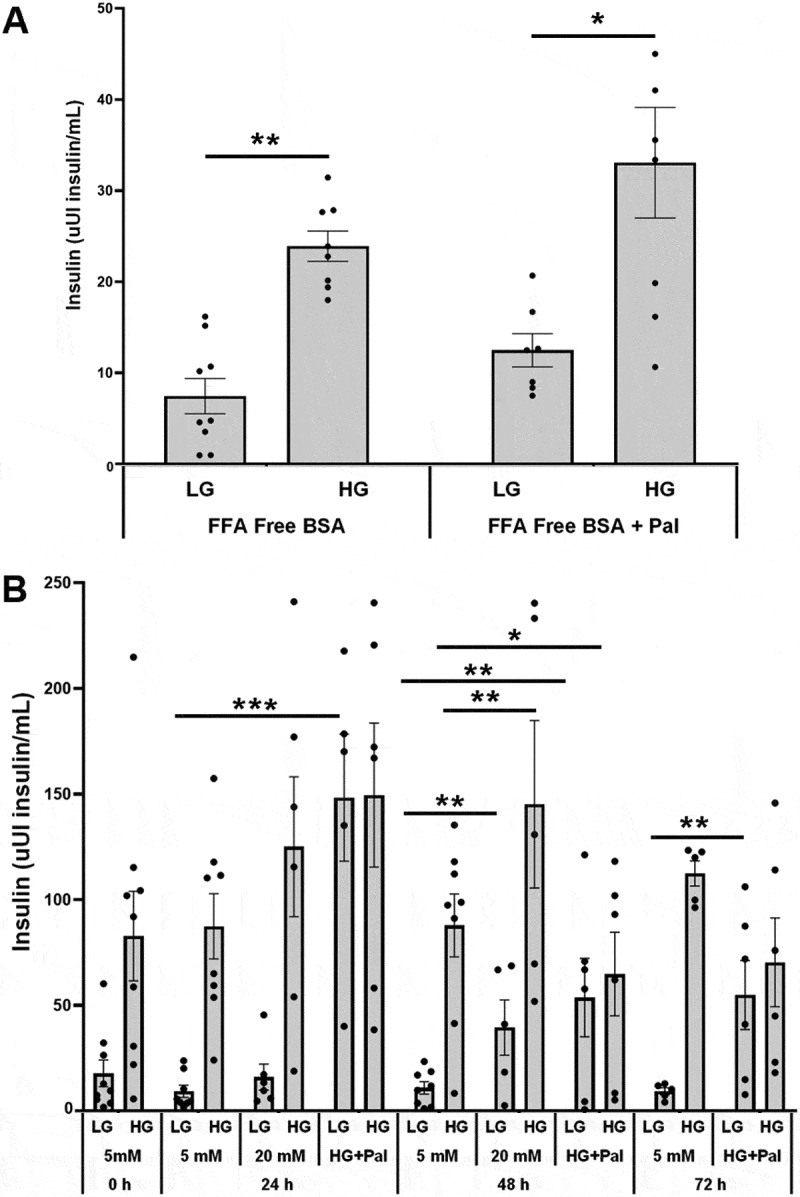
A) The effects of sodium palmitate on glucose-stimulated insulin secretion in human islets. Islets were pretreated with 0.31% FFA-free BSA for 1 hour. Islets were then treated with 0.31% FFA free BSA with 2 mM low glucose (LG) or 8 mM high glucose (HG) plus or minus 400 µM palmitate (*n* = 6–8). B) The chronic effects of treating human islets with high glucose plus or minus sodium palmitate on glucose-stimulated insulin secretion. Islets were cultured with RPMI1640 containing 5 or 20 mM glucose plus or minus 200 µM sodium palmitate for 0 to 72 hours. After culturing islets, an insulin secretion assay was performed by first pretreating islets with 0.1% regular BSA for 1 hour. Islets were treated with 0.1% regular BSA with 2 mM low glucose (LG) or 16.7 mM high glucose (HG). (*n* = 4–9). After the insulin secretion assay, islets were collected, and ARNT gene expression was assessed (*n* = 3–4). *p < 0.05, **p < 0.01, ***p < 0.001.

Human islets were pretreated with 0.31% FFA-free BSA for 1 hour. Islets were then acutely treated with 0.31% FFA-free BSA with LG or 8 mM high glucose (HG) plus or minus 400 µM palmitate. Sodium palmitate was prepared using method 2. Incubating human islets with HG with or without palmitate significantly increased insulin secretion compared to LG (Figure 9A). At LG or HG levels, acute palmitate did not significantly increase insulin secretion compared to LG or HG alone respectively (Figure 9A).

The chronic effects of treating human islets with high glucose plus or minus sodium palmitate on glucose-stimulated insulin secretion were assessed next. These studies cultured islets with 5 or 20 mM glucose plus or minus 200 µM sodium palmitate for 0 to 72 hours. Sodium palmitate was prepared using method 2. Chronic treatment with 20 mM glucose increased basal insulin secretion over 48 hours (Figure 9B). Chronic treatment of human islets with both 20 mM glucose and 200 µM sodium palmitate significantly increased basal insulin secretion and initially increased, followed by reduced high glucose-stimulated insulin secretion over 72 hours (Figure 9B).

## Discussion

The ability of FFAs to stimulate insulin secretion from β-cells is an important aspect of the postprandial response to a meal. However, the mechanisms governing FFA delivery to β-cells remain incompletely understood. A key concept is that only free, unbound FFAs influence β-cell function, and their levels are regulated by the FFA-to-BSA ratio, with higher ratios yielding more unbound FFAs. The ability to assess the effects of FFA *in vitro* is complicated by the variable methods of FFA preparation and how they are incubated with cells. This study demonstrates that using FFA-free BSA as a control can independently affect β-cell function. Additionally, we show that the method of FFA preparation significantly influences FFA solubility and its effects on insulin secretion in both clonal β-cells and human islets.

These studies show that increasing FFA-free BSA concentrations in the assay buffer dose-dependently inhibited insulin secretion in the clonal β-cells 832/13 cells. However, preincubation of cells with FFA-free BSA followed by incubating cells with regular BSA did not affect insulin secretion to the same degree. This suggests that FFA-free BSA primarily affects insulin secretion during the treatment period and that FFA-free BSA may deplete cells of a critical factor(s) that are important for regulating insulin secretion. This factor(s) is currently unknown, and our laboratory is investigating its identity. Some potential signaling molecules could be affected by FFA-free BSA, including 1-monoacyl.^35,48^ These studies also suggest that using FFA-free BSA as a control for insulin secretion assays may be problematic.

These studies show that palmitate can enhance glucose-stimulated insulin secretion in 832/13 cells compared to cells incubated with FFA-free BSA. These results are consistent with previous publications on the effects of palmitate on insulin secretion.^35,48–50^ However, the effects of palmitate depend on the method of palmitate preparation. When palmitate was prepared with method 1, the ability of palmitate to potentiate insulin secretion was not apparent or not as strong as compared to cells incubated with regular BSA. In contrast, its effects were more apparent when palmitate was prepared with method 2. Method 1 was not as effective at dissolving palmitate compared to method 2. When the measured palmitate concentrations were matched, the palmitate method 1 still did not lead to a strong potentiation of insulin secretion compared to method 2. These studies also show that the ratio of palmitate to BSA significantly affects palmitate’s ability to potentiate insulin secretion, consistent with previous publications.^35,48–50^

Changing the palmitate to BSA ratio affects the unbound palmitate concentration and palmitate’s effectiveness in stimulating insulin secretion. The unbound FFA concentration close to β-cells is unknown, but in human plasma, it is estimated to have between 5 nM and 50 nM of unbound FFAs.^7,51,52^ BSA is commonly used for *in vitro* islet studies to improve solubility and control unbound fatty acid concentrations when FFA is used as a stimulus. Since regular BSA may contain variable amounts of lipids and other metabolites, FFA-free BSA is often used to control the exact composition of lipids during an islet experiment.^10,53^ Results presented here suggest that FFA-free BSA present a problem as a control. There are also different ways to prepare FFA-free BSA, which may complicate matters further. FFA-free BSA can be purchased from a company such as SIGMA or prepared by treating BSA with charcoal. BSA treated with charcoal will allow the removal of some contaminants, including lipids.^53–58^ Commercially available FFA-free BSA typically has higher purity and less residual fatty acids than charcoal-absorbed BSA.^53–58^

Since the method of FFA preparation affects the measured concentration of FFA, it may also affect the unbound concentration of FFA. In the few studies that report unbound FFA concentrations, a multiple-stepwise equilibrium model is used to calculate an estimate of the unbound FFA concentrations using the molar ratio of FFA: BSA.^54,59^ A more accurate measure of unbound FFA concentrations is to measure it using a fluorescent probe ADIFAB2, which is an acrylodan-derivatized intestinal fatty acid binding protein.^10,16,17,60–62^ The problem with calculating unbound FFAs is that it does not consider the method used to complex FFAs to albumin. For example, the same unbound concentration of FFA can be measured when FFAs are prepared with different concentrations, ratios of FFA to BSA and forms of albumin, such as 1% charcoal-absorbed BSA, 0.75% commercial FFA-free BSA or pre-complexed FFAs used in the presence of 0.67% FFA-free BSA.^16,54^ Another problem with estimating unbound FFA concentrations is that albumin has different affinities for FFAs that depend on the carbon length and degrees of saturation. For example, palmitate, oleate, stearate and arachidonate have different affinities for albumin and thus, similar FFA: BSA ratios for these lipids will have different unbound FFA concentrations.^59^ In one study, it was shown using ADIFAB2, the unbound FFA was about 26 nM for palmitate and 35 nM for oleate in a 1% charcoal-absorbed BSA at an FFA/albumin molar ratio of 3.3:1 which is different from the calculated estimate of unbound FFA concentrations.^16^

These studies show that increasing the glucose concentration can significantly elevate the glycolytic rate, whereas there was only a slight increase in oxygen consumption. These results are consistent with recent evidence indicating that ATP derived from the glycolytic enzyme pyruvate kinase is more critical for regulating K_ATP_ channels in β-cells.^63–66^ These studies show that palmitate can increase the oxygen consumption rate. The Seahorse pH-sensitive probe determines the ECAR to estimate the glycolytic rate. Unfortunately, unlike the Seahorse oxygen-sensitive probe, the ECAR probe was affected by changes in the BSA concentration and did not allow the assessment of the effects of palmitate on the glycolytic rate. These data indicate that palmitate can enhance glucose-stimulated oxygen consumption rate, which is best at an intermediate glucose concentration.

Using method 2 to prepare palmitate along with the high palmitate to BSA ratio that strongly stimulated insulin secretion in the 832/13 cells did not significantly increase insulin secretion in human islets. There was a trend for increased insulin secretion with palmitate, consistent with previous publications.^3,11,37^ We also assessed the effects of chronically treating human islets with high glucose plus or minus sodium palmitate on glucose-stimulated insulin secretion. Chronic treatment with high glucose alone increased basal insulin secretion, whereas incubating with both high glucose and palmitate initially significantly increased basal insulin secretion, followed by reduced high glucose-stimulated insulin secretion.

When performing islet lipid studies, an important question is what concentrations should be used and how lipids change close to islets after a meal. The changes in lipid profiles during a meal are complicated and involve a switch from the fasted to fed state, which inhibits lipolysis from adipocytes and leads to reduced plasma lipid metabolites such as glycerol, FFA and acylcarnitine by about 50–70% postprandially.^67^ An assumption in islet research is that there is a postprandial rise in plasma FFA close to islets due to gut absorption. In fact, there is a drop in postprandial plasma FFA levels primarily due to insulin-mediated inhibition of adipocyte lipolysis.^68,69^ Most FFA absorbed in the gastrointestinal tract are packaged as TGs inside of chylomicrons, and any changes in plasma FFA postprandially are likely due to spill-over during transport.^70,71^ For example, when a standard Western diet or mixed test meal is given, there is a slow drop in human plasma FFA to 200 μmol/L after about 90 minutes postprandial, followed by a slow rise to 800 μmol/L after 360 minutes. The slow rise of plasma FFA postprandially is due to the switch back to a fasting state.^68,69^ Total plasma triglycerides go from about 1000 μmol/L to a peak of 2000 μmol/L postprandially after about 3 hours.^68,69,72–80^ These studies suggest lipid experiments in islets should be done starting with elevated FFA followed by dropping the FFA when islets are stimulated with high glucose, as seen in humans postprandially.

Overnight fasting FFA levels is about 475 μmol/L in human blood plasma.^81^ Fasting for 24 hours can increase plasma FFAs to about 1000 μmol/L and 2000 μmol/L after 48 hours of fasting.^35,71^ Since the typical fasting duration is overnight, a good starting point for control islet experiments is to use a total FFA concentration of around 475 μmol/L. Palmitate accounts for only about 23% of the total FFA concentration, which suggests that fasting palmitate concentrations in plasma are around 109 μmol/L.^81–85^ However, most control islet lipid experiments are done without any FFA, and they often use FFA-free BSA, which can pull lipids out of islets.^18–27,34–37^ In the current studies, palmitate was effective at increasing insulin secretion above 200 μmol/L with a high palmitate to BSA ratio, which may not be physiological.

Some questions arise from the current set of studies. For example, when treating cells with FFAs, do we treat control cells with no FFA, or do we need to balance the total amount of lipids when assessing the effects of a single FFA such as palmitate? Another consideration is whether we control the lipid concentration, BSA concentration, and unbound FFA concentration or the ratio of FFA to BSA. If we use palmitate and conjugate it to albumin, do we use a lower albumin concentration to be sure we have an appropriate level of unbound palmitate, or do we conjugate palmitate to a physiological concentration of albumin and use another lipid to balance the total FFA in the preparation? As shown in this paper, using a lower albumin concentration or different forms of albumin will complicate the interpretation of the results. These issues are not easily controlled for *in vitro* islet experiments and likely require multiple controls. Although more work is needed, we recommend using regular and FFA-free BSA as controls in islet lipid studies.

Other important caveats should be considered: Should the albumin concentration be what is found in plasma or the interstitial space? What is the islet exposure to FFA postprandially? Are local FFA near islets dependent on changes in plasma FFA, the delivery of FFA from changes in lipoprotein lipase activity on or near islets that release FFA from TG stores in chylomicrons and VLDLs or FFAs released by islets themselves from internal stores.^33,86^ It is recommended that to compare islet lipid studies, the method of lipid preparation, whether a concentrated stock of FFA was used, the time from preparation to use in experiments, was FBS used, what media/buffer was used for the experiments, what controls are used, and FFA/albumin molar ratio should be reported. Since the local unbound concentration of FFA is important in controlling cellular uptake^87^ and can potentiate glucose-stimulated insulin secretion,^9^ the unbound FFA concentration should also be measured.

## Supplementary Material

Supplemental table.docx

## Abbreviations

CATR: Carboxyatractyloside
BSA: Bovine serum albumin
ECAR: Extracellular acidification rate
FFA: Free fatty acids
FFA free BSA: Free fatty acid-free bovine serum albumin
HG: High glucose
LG: Low glucose
MG: Medium glucose
OCR: Oxygen consumption rate
