# Supplementary material for: The effects of free fatty acid-free bovine serum albumin and palmitate on pancreatic β-cell function
Source: Islets. 2025 Mar 16;17(1):2479911. doi: 10.1080/19382014.2025.2479911 (PMC11917175; doi:10.1080/19382014.2025.2479911)
Supplement: Supplemental table.docx [file KISL_A_2479911_SM9772.docx]

**The effects of free fatty acid-free bovine serum albumin and palmitate on pancreatic β-cell function.**

Katherine Sentjens^1^, Renjitha Pillai^1^, Jamie W. Joseph^1*^

^1^University of Waterloo, School of Pharmacy, Kitchener, Ontario, Canada

* Address correspondence to:

School of Pharmacy; Health Science Campus; Room 4008

University of Waterloo; 10A Victoria Street South

KITCHENER, Ontario; N2G 1C5

Supplemental Table:

**Supplementary Table 1:** Human organ donor information. The Alberta Diabetes IsletCore provided the human islets in 2012-2013. BMI - Body Mass Index, T2D - Type 2 Diabetes, NP – not provided.

| **Donor ID** | **BMI** | **Age** | **Biological Sex** | **T2D Status** |
| --- | --- | --- | --- | --- |
| \| R038 \| \| --- \| | NP | 51 | Male | No |
| R039 | NP | 46 | Male | No |
| R042 | NP | 44 | Male | No |
| R043 | NP | 60 | Male | No |
| R044 | NP | 38 | Male | No |
| R047 | NP | 56 | Male | No |
| R049 | NP | 79 | Male | No |
| R050 | NP | 72 | Male | No |
| R051 | NP | 73 | Male | No |
